# Intermittent Hypoxia Activates Duration-Dependent Protective and Injurious Mechanisms in Mouse Lung Endothelial Cells

**DOI:** 10.3389/fphys.2018.01754

**Published:** 2018-12-06

**Authors:** Peter Wohlrab, Lourdes Soto-Gonzales, Thomas Benesch, Max Paul Winter, Irene Marthe Lang, Klaus Markstaller, Verena Tretter, Klaus Ulrich Klein

**Affiliations:** ^1^Department of Anaesthesia, General Intensive Care and Pain Management, Medical University of Vienna, Vienna, Austria; ^2^Institute for International Development, University of Vienna, Vienna, Austria; ^3^Department of Internal Medicine II, Medical University of Vienna, Vienna, Austria

**Keywords:** lung endothelium, intermittent hypoxia, obstructive sleep apnea, alternating oxygen conditions, atelectasis

## Abstract

Intermittent hypoxia is a major factor in clinical conditions like the obstructive sleep apnea syndrome or the cyclic recruitment and derecruitment of atelectasis in acute respiratory distress syndrome and positive pressure mechanical ventilation. *In vivo* investigations of the direct impact of intermittent hypoxia are frequently hampered by multiple co-morbidities of patients. Therefore, cell culture experiments are important model systems to elucidate molecular mechanisms that are involved in the cellular response to alternating oxygen conditions and could represent future targets for tailored therapies. In this study, we focused on mouse lung endothelial cells as a first frontier to encounter altered oxygen due to disturbances in airway or lung function, that play an important role in the development of secondary diseases like vascular disease and pulmonary hypertension. We analyzed key markers for endothelial function including cell adhesion molecules, molecules involved in regulation of fibrinolysis, hemostasis, redox balance, and regulators of gene expression like miRNAs. Results show that short-time exposure to intermittent hypoxia has little impact on vitality and health of cells. At early timepoints and up to 24 h, many endothelial markers are unchanged in their expression and some indicators of injury are even downregulated. However, in the long-term, multiple signaling pathways are activated, that ultimately result in cellular inflammation, oxidative stress, and apoptosis.

## Introduction

In the human body, the pulmonary circulation has a unique function as it receives total cardiac output to provide gas exchange of carbon dioxide (CO_2_) for oxygen (O_2_). Special issues of the pulmonary vascular bed are the maintenance of a low vascular resistance, despite the exposure to mechanical stress, and to the highest O_2_ tension in the body (Suresh and Shimoda, 2016). The cellular monolayer of the pulmonary endothelium plays a prime role as to the functioning of the healthy lung with regard to proper perfusion and unimpaired gas exchange. It regulates coagulation, interaction with blood and immune cells, maintains a crucial barrier function and produces and interacts with vasoactive mediators like nitric oxide (NO), endothelin, thromboxane, serotonin, prostacyclin, and other signaling molecules like cytokines. Many lung diseases like pulmonary hypertension (PH) or acute respiratory distress syndrome (ARDS) develop due to endothelial dysfunction.

The lung endothelium is actually exposed to the lowest and highest O_2_ tension compared to other vascular beds in the body. Blood with high CO_2_ content and a partial pressure of O_2_ (pO_2_) of approximately 40 mmHg, derived from the right ventricle passes the precapillary pulmonary circulation and blood with a pO_2_ of approximately 100 mmHg in the postcapillary segment returns to the left atrium of the heart. The ultrastructure of endothelial cells in the different segments varies according to the respective function. Under hypoxic conditions, the pulmonary vasculature undergoes a meanwhile broadly investigated hypoxia-induced remodeling including vasoconstriction that might lead in its chronic form to pulmonary hypertension (Thompson and Lawrie, 2017).

Less well understood are effects of intermittent hypoxia on the lung endothelial cells. The condition of intermittent hypoxia has several causes. It is a hallmark of obstructive sleep apnea syndrome (OSAS), but is also encountered in cyclic recruitment and derecruitment of atelectasis (CA) during positive pressure mechanical ventilation. OSAS is *per definitionem* a full syndrome including sympathetic activation, sleep fragmentation, and is frequently associated with obesity, advanced age, and co-morbidities. A direct and isolated effect of intermittent hypoxia on different cell types can best be initially investigated in cell culture. This is of importance especially in the light of recent scientific discussions as to whether intermittent hypoxia *per se* has a detrimental, or on the contrary, a protective impact on different cell types of the vascular system (Lavie and Lavie, 2017).

In our study, we aimed to analyze cell responses of endothelial cells from mouse lung to short-term and chronic intermittent hypoxia with regard to cell growth, gene expression of key endothelial markers, redox systems, and regulatory molecules (miRNAs). We show that depending on exposure time, intermittent hypoxia can have both, protective and injurious effects on lung endothelial cells.

## Materials and Methods

### Ethical Considerations

Isolation of endothelial cells from mouse lungs was performed after animals have been humanely killed according to the current legislation (Austrian Animal Experiment Law 2012). Permission for these experiments was granted by the local animal ethics committee at Medical University Vienna and the Federal Ministry for Science, Research and Economy of Austria (GZ: BMWFW-66.009/0089-WF/v/3b/2016).

### Isolation of Murine Lung Endothelial Cells and Cell Culture

Mouse primary lung endothelial cells were isolated from enzymatically digested lung tissue from adult C57B/L6 mice by magnetic separation, according to the method described in Zirlik et al. (2007). Cells were plated on dishes coated with 2% gelatine and 10 μg/ml fibronectin (Sigma-Aldrich, United States; cat.nr. F1141) in M199 medium (ThermoFisher, Waltham, MA, United States; cat.nr. 41150-020), 20% fetal calf serum superior (Biochrom GmbH, Germany; cat.nr. S0615), endothelial cell growth supplement from bovine pituitary (Sigma-Aldrich, United States; cat.nr. E0760), 5 U/ml heparin, and antibiotics (penicillin/streptomycin: ThermoFisher; Waltham, MA, United States; cat.nr. 15140-122; amphotericin: ThermoFisher, Waltham, MA, United States; cat.nr. 15290-026).

### Exposure of Cells to Different Oxygen Conditions

Three weeks after isolation the expanded cultures were used for experiments. Cells were trypsinized and plated into six-well plates containing gas-permeable membranes (imaging plates from Zellkontakt, Nörten-Hardenberg, Germany; cat.nr. 3221-20). Twenty-four hours after plating adherent cells were transferred to custom-made boxes [as described in Hafner et al. (2016)] and exposed to four different O_2_ conditions supplied by premixed gas bottles: (1) 21% O_2_ – 5% CO_2_ – 74% N_2_; (2) 0% O_2_ – 5% CO_2_ – 95% N_2_; (3) 10% O_2_ – 5% CO_2_ – 85% N_2_; (4) 0–21% O_2_ oscillations – 5% CO_2_ – rest N_2_ with a frequency of six oscillations per hour [as described in Wu et al. (2016)]. Analysis of cell responses was performed after 4, 24, and 72 h of exposure.

### Analysis of Cell Mass

To quantify cell numbers after 4, 24, and 72 h of gas exposure we counted cells from individual wells (a total of 6 wells/condition) in a Neubauer chamber after staining with trypan blue.

### Analysis of Cytokine Release

Secreted cytokines/chemokines (IL6; CXCL1; MIP2) were analyzed in cell culture supernatants by ELISA using Duoset ELISA kits (R&D Systems, MN, United States: IL6: cat.nr. DY406; CXCL1/KC: cat.nr. DY453; CXCL2/MIP2: cat.nr. DY452) and were normalized to cell numbers at the respective time point.

### Analysis of Changes in Gene Expression of Key Functional Endothelial Markers

Changes in gene expression were analyzed by quantitative real-time PCR. Total RNA was isolated with a RNAeasy plus kit (Qiagen, Netherlands; cat.nr. 74136). One nanogram of mRNA was reverse transcribed using qScript cDNA synthesis kit (Quanta Biosciences; cat.nr. 95048) and resulting cDNA was analyzed on a RotorGene Q cycler (Qiagen) using PerfeCTa SYBR^®^ Green FastMix (Quanta Biosciences, MD, United States; cat.nr. 95072-012). For primer sequences, see Table 1. The cycling program comprised 30 s 95°C for denaturing, and 45 cycles of 5 s 95°C, 15 s 55°C, and 10 s 72°C. Expression of beta-actin was used for internal normalization.

**Table 1:** Primer sequences for qRT-PCR.

Gene	Sequence	Gene	Sequence
*Vegfa*	5′-AATGCTTTCTCCGCTCTGAA-3′ 5′-GATCATGCGGATCAAACCTC-3′	*Angpt2*	5′-GATCTTCCTCCAGCCCCTAC-3′5′-TTTGTGCTGCTGCTGTCTGGTTTC-3′
*TNFα*	5′-CATCTTCTCAAAATTCGAGTGACAA-3′ 5′-TGGGAGTAGACAAGGTACAACCC-3′	*Selplg* (p-selectin)	5′-GAACAATCCAGGTTGCCTTG-3′ 5′-CAGTTCATGTGCGATGAAGG-3′
*Vwf*	5′-CCGTCTTCAGTAGCTGGCAT-3′ 5′-CAGACCCCTATGATTTTGCC-3′	*Vcam1*	5′-ACCAAGGAAGATGCGCAGTA-3′ 5′-CCGGCATATACGAGTGTGAA-3′
*Ace*	5′-CGGAAAGTCACGGGTAAGTC-3′ 5′-GGACTTCTACAACGGCAAGG-3′	*Icam1*	5′-AAGGTGGTTCTTCTGAGCGG-3′ 5′-TCCAGCCGAGGACCATACAG-3′
*Plau* (uPA)	5′-GTGTAGACACCGGGCTTGTT-3′ 5′-GGACCCAGAGTGGAAAACAG-3′	*Rela* (NFκB p65)	5′-CATAGGTCCTTTTGCGCTTC-3′ 5′-AGGCTCCTGTTCGAGTCTCC-3′
*Plat* (tPA)	5′-TGAACCTCCCATGTATTCCC-3′ 5′-CCTGCAAGAGTGGGAAAGAA-3′	*Hmox1*	5′-CAGAAGAGGCTAAGACCGCC-3′ 5′-CTCTGCAGGGGCAGTATCTTG-3′
*Serpine1* (PAI1)	5′-ACTGTCCTATCTCAAGGTCC-3′ 5′-TGATCTGTCTATCCGTTGCCC-3′	*Nos3*	5′-GTTGTACGGGCCTGACATTT-3′ 5′-GGTCCTGTGCATGGATGAG-3′
*Ager* (RAGE)	5′-AACACAGGAAGAACTGAAGCTTGG-3′ 5′-CTTTGCCATCGGGAATCAGAAGTT-3′	*Actb (reference gene)*	5′-AGCCATGTACGTAGCCATC-3′ 5′-CTCTCAGCTGTGGTGGTGA-3′


### Analysis of Changes in Gene Expression of Redox Systems

In order to analyze the effects of different O_2_-conditions on the cellular proteinous redox systems, we used the mouse oxidative stress PCR array from SABiosciences (Qiagen, Netherlands; cat.nr. PAMM-065Z). The array features 84 genes involved in the response to oxidative stress and reactive O_2_ metabolism as well as oxygen transporters. For information on the full list of tested genes, see the Supplementary Figure S1. Beta-2-microglobulin (*B2M*) was used for normalization.

### Analyses of Protein Levels by Western Blotting

Cells were lysed after 4, 24, and 72 h of gas exposure in RIPA buffer [25 mM Tris/HCl pH 7.5; 150 mM NaCl; 0.1% SDS; 0.5% sodium deoxycholate; 1% NP-40; complete mini protease inhibitor tablet (Roche; cat.nr. 11836153001)]. Equal amounts of proteins as analyzed by BCA protein assay (Thermo Scientific; Waltham, MA, United States) were loaded on an 8, 10, or 12% SDS-PAGE gel. After separation, proteins were semidry-blotted onto nitrocellulose membranes. Specific bands were detected with the following antibodies: anti-ICAM1 (R&D Systems cat.nr. AF796; 1:10.000); anti-VCAM1 (R&D Systems cat.nr. AF643; 1:4000); anti-p65NFκB (R&D Systems cat.nr. MAB5078; 1:250); anti-SOD2 (R&D Systems; cat.nr. MAB3419; 1:2000); anti-TXN1 (CST; cat.nr. #2298; 1:1000); anti-HMGB1 (CST; cat.nr. #3935; 1:1000); and anti-ß-actin (Santa Cruz; cat.nr. sc-130656; 1:1000). For analysis of phosphorylated ERK1/2 cell lysates were prepared in the presence of a protease and phosphatase inhibitor mini tablet (PIERCE; Thermo Scientific; cat.nr. A32959). The primary antibodies were as follows: anti-phospho ERK1/2 (CST; cat.nr. #9106; 1:2000) and anti-ERK1/2 (CST; cat.nr. #9102). After incubation with primary antibodies overnight at 4°C, the washed membranes were incubated with the respective HRP-coupled secondary antibodies for 45 min at room temperature. The chemiluminescence reaction was developed with Westernbright Sirius reagent (Biozym; Hess.Oldendorf, Germany) and analyzed on a Vilber Lourmat (Eberhardzell, Germany) imaging system.

### Analysis of Expression Levels of miRNAs

For miRNA expression profiling we used the miScript miRNA PCR Array Mouse miFinder (Qiagen, Netherlands; cat.nr. 331221 MIMM-001ZR). For information on the full list of tested miRNAs, see the Supplementary Figure S2. After isolating miRNAs using the miRNeasy mini kit (Qiagen, Netherlands; cat.nr. 217004), the miRNAs were polyadenylated and amplified using a sequence specific primer in addition to an universal primer for qRT-PCR.

### Data Analysis of qRT-PCR Analysis

Changes in gene expressions under different exposure conditions (relative to control: 21% O_2_) were expressed as “fold change” and were analyzed by the comparative Ct (ΔΔCt) method.

### Analysis of Endothelial Monolayer Permeability

We used a method described by Dubrovsky et al. (2013) to quantify local permeability of pulmonary endothelial cell monolayers. Mouse lung endothelial cells were isolated and plated on gas-permeable 96-well plates (Zellkontakt, Nörten-Hardenberg, Germany; cat.nr. 3241-20) coated with biotinylated gelatine. For biotinylation gelatine from porcine skin (Sigma-Aldrich cat.nr. G2500) was dissolved in 0.1 M NaHCO_3_ pH 8.3 at 10 mg/ml in a 70°C water bath. After centrifugation, the gelatin was combined with previously dissolved EZ link NHS-LC-LC-biotin (Thermo Scientific, Rockland, IL, United States; cat.nr. 21343) in DMSO (5.7 mg/ml stock solution) at a final concentration of 0.57 mg/ml and incubated at room temperature for 1 h on a rotator. Dishes were coated with biotinylated gelatin at a concentration of 0.25 mg/ml (50 μl/well) overnight at 4°C. Plates were washed two times with 200 μl PBS and seeded with 3 × 10^4^ cells/well in full serum-containing M199 medium. Cells were grown in a normal incubator 72 h prior to gas exposure. After 4, 24, and 72 h of exposure to gas conditions as described above, FITC-avidin (Thermo Fisher Scientific cat.nr. 434411) was added to the culture medium at a final concentration of 25 μg/ml for 3 min. Unbound FITC-avidin was washed out with 2 × 200 μl PBS. Finally, 100 μl PBS were added and fluorescence was measured on a Victor3 Plate reader (Perkin Elmer, Waltham, MA, United States) using Ex = 485 nm and Em = 535 nm; 0.1 s, at the bottom.

### Study Design and Statistical Analysis

**FIGURE 1:**
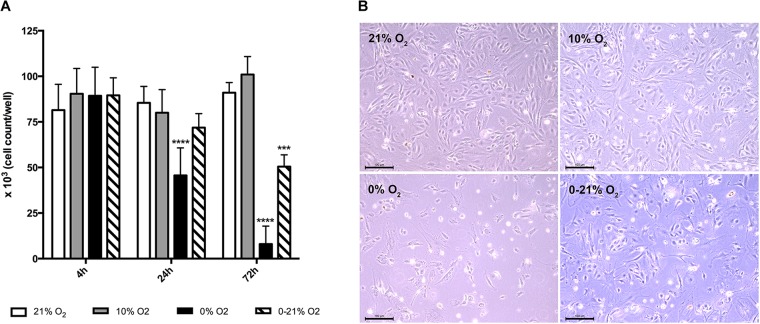
Cell viability under short-term and chronic exposure to different oxygen conditions. **(A)** Total live cell numbers per well (six-well plate) were counted after trypan blue staining and compared to normoxia. ^∗∗∗^*p* < 0.001; ^∗∗∗∗^*p* < 0.0001. **(B)** Phase contrast microscopy of cells after 72 h exposure to different oxygen conditions. Scale bar: 100 μm.

**FIGURE 2:**
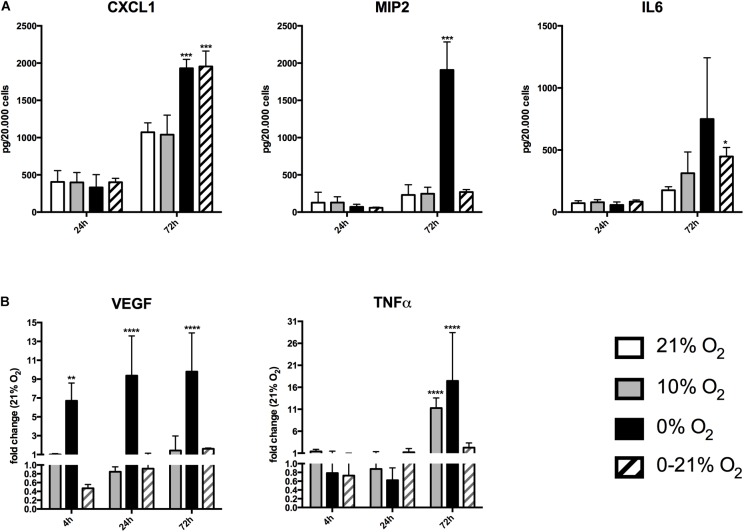
Expression and release of chemokines/cytokines in response to short-term and chronic exposure to different oxygen conditions. **(A)** Release of CXCL1, MIP2, and IL6 was quantified in cell culture supernatants by ELISA and was normalized to cell numbers. ^∗^*p* < 0.05; ^∗∗∗^*p* < 0.001. **(B)** Relative expression of intracellular mRNA for VEGF and TNFα after short-term and chronic exposure to different oxygen conditions. ^∗∗^*p* < 0.01; ^∗∗∗∗^*p* < 0.0001.

All experiments were conducted in at least *n* = 6 replicates and were repeated three times, each with a new cell preparation. Exceptions are oxidative stress and miRNA arrays as well as Western blot experiments with *n* = 3. Mean values in the text are given as mean ± standard deviation (SD). We used SPSS^®^ software (SPSS Version 24) for statistical analysis. Statistic tests for comparison of multiple groups (Figures 1, 2A) were a two-way ANOVA (Bonferroni correction), for comparison of two groups, we used Student’s *t*-test. *P*-values below 0.05 were regarded as statistically significant.

## Results

### Experimental Set-Up

Primary mouse lung endothelial cells were isolated, grown for 2 weeks and repurified by magnetic separation in order to prevent contamination with other cell types. Cells were plated at a density of 1–2 × 10^5^ cells /well in a six-well plate and after 24 h were exposed to four different gas conditions: normoxia (21% O_2_, 5% CO_2_, 74% N_2_), moderate/intermediate hypoxia (10% O_2_, 5% CO_2_, 85% N_2_), anoxia (0% O_2_, 5% CO_2_, 95% N_2_), intermittent hypoxia/O_2_ oscillations (0–21% O_2_, 5% CO_2_, rest N_2_) at a frequency of six cycles per hour. At different timepoints, we evaluated cell growth, cytokine release, gene and protein expression, and monolayer permeability. For permeability assays, cells were plated in 96-well plates as described in the Materials and Methods section and exposed to the same gas conditions.

### Chronic Intermittent Hypoxia Induces Cell Death in Primary Lung Endothelial Cells

As a measure for living cell mass, we used trypan blue staining and counted white cells/well in a Neubauer Chamber (Figure 1A). After 4 h, the mean was 81.5 ± 14.1 × 10^3^ under normoxia, 90.4 ± 13.9 × 10^3^ under moderate hypoxia, 89.3 ± 15.6 × 10^3^ under anoxia, and 89.5 ± 9.5 × 10^3^ under intermittent hypoxia. At 24 h, cell numbers were 85.4 ± 9.0 × 10^3^ under normoxia, 80.0 ± 12.7 × 10^3^ under moderate hypoxia, 45.6 ± 15.1 × 10^3^ under anoxia, and 71.8 ± 7.6 × 10^3^ under intermittent hypoxia indicating a loss of 47% for anoxia compared to normoxia (*p* < 0.0001). At 72 h, cell numbers were 91.0 ± 5.6 × 10^3^ under normoxia, 101.0 ± 9.8 × 10^3^ under moderate hypoxia, 8.0 ± 9.8 × 10^3^ under anoxia, and 50.5 ± 6.5 × 10^3^ under intermittent hypoxia indicating a gross loss of living cells under anoxia and a reduction by 45% under intermittent hypoxia (*p* < 0.001 versus normoxia). Figure 1B exhibits representative microscopic phase contrast images after 72 h showing also morphological changes under hypoxic conditions.

### Chronic Intermittent Hypoxia and Anoxia Induce Inflammatory Cytokines

We measured the release of the chemokines/cytokines (C-X-C motif) ligand 1 (CXCL1, KC), macrophage inflammatory protein 2 (MIP2), and interleukin 6 (IL6) into the culture supernatants under all four different O_2_ conditions (Figure 2A). Normalized values (relative to cell numbers at the respective time points) of CXCL1 after 24 h were 405 ± 151 pg under normoxia, 396 ± 134 pg under moderate hypoxia, 331 ± 172 pg under anoxia, 401 ± 52 pg under intermittent hypoxia. After 72 h, CXCL1 levels raised to 1071 ± 126 pg under normoxia, 1038 ± 261 pg under moderate hypoxia, 1930 ± 120 pg under anoxia, and 1954 ± 207 pg under intermittent hypoxia. This indicates an 80% and 82% increase of CXCL1 levels under anoxia and intermittent hypoxia, respectively, compared to normoxia (*p*-values: 0.018 and 0.043, respectively). Normalized MIP2 levels after 24 h were 125 ± 139 pg under normoxia, 128 ± 78 pg under moderate hypoxia, 71 ± 34 pg under anoxia, and 58 ± 8 pg under intermittent hypoxia. After 72 h, MIP2 levels had increased to 229 ± 135 pg under normoxia, 246 ± 87 pg under moderate hypoxia, 1907 ± 377 pg under anoxia (*p*-value: 0.012 versus normoxia), but only 269 ± 34 pg under intermittent hypoxia.

After 24 h, IL6 levels were 72 ± 19 pg under normoxia, 79 ± 21 pg under moderate hypoxia, 58 ± 24 pg under anoxia, and 84 ± 14 pg under intermittent hypoxia. After 72 h, IL-6 levels increased to 176 ± 28 pg under normoxia, 313 ± 171 pg under moderate hypoxia, 749 ± 493 pg under anoxia, and 447 ± 72 pg under intermittent hypoxia. Therefore, IL6 levels were increased by 78% under moderate hypoxia, 325% with high variation (due to the low number of remaining cells) under anoxia, and 154% under intermittent hypoxia compared to normoxia. The value of intermittent hypoxia reached statistical significance (*p* = 0.043).

We further assessed VEGF and TNFα expression as cellular mRNA levels by qRT-PCR, as secretion into the cell culture supernatant was not detectable under basal conditions (Figure 2B). Using normoxia as reference, VEGF fold regulation after 4 h was +1.1 ± 0.08 under moderate hypoxia, +6.7 ± 1.9 under anoxia (*p* < 0.01) and 0.5 ± 0.08 under intermittent hypoxia. Fold change after 24 h was 0.8 ± 0.1 under moderate hypoxia, +9.4 ± 4.2 under anoxia (*p* < 0.0001) and 0.9 ± 0.2 under intermittent hypoxia. After 72 h, VEGF fold regulation compared to normoxia was +1.4 ± 0.1 under moderate hypoxia, +9.8 ± 4.1 (*p* < 0.0001) under anoxia, and +1.61 ± 0.1-fold under intermittent hypoxia.

TNFα fold regulation after 4 h was +1.4 ± 0.5 under moderate hypoxia, 0.8 ± 0.7 under anoxia, and 0.7 ± 0.3 under intermittent hypoxia. At 24 h fold change was 0.9 ± 0.4 under moderate hypoxia, 0.6 ± 0.3 under anoxia, and +1.2 ± 0.8 under intermittent hypoxia. After 72 h, TNFα fold regulation compared to normoxia was +11.3 ± 2.2 under moderate hypoxia (*p* < 0.0001), +17.5 ± 10 with high variation under anoxia (*p* < 0.0001), and +2.2 ± 1.0 under intermittent hypoxia.

### Short-Term and Chronic Intermittent Hypoxia Reveals Different Gene Expression Patterns of Key Endothelial Markers

**FIGURE 3:**
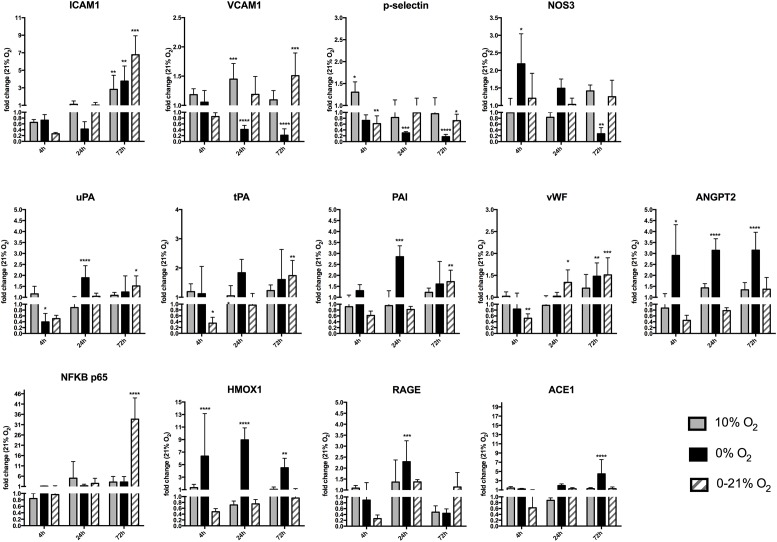
Gene expression of different endothelial cell functional marker proteins after short-term and chronic exposure to different oxygen conditions. Cell adhesion molecules ICAM1, VCAM1, and p-selectin, endothelial nitric oxide synthase 3 (NOS3), urokinase-type plasminogen activator (uPA), tissue plasminogen activator (tPA), plasminogen activator inhibitor-1 (PAI1), Von Willebrand factor (vWF), angiopoietin-2 (ANGPT2), nuclear factor NF-kappa-B (NFκB p65), heme oxygenase-1 (HMOX1), receptor of advanced glycation endproducts (RAGE), angiotensin-converting enzyme-1(ACE1). Relative gene expression was quantified by qRT-PCR using the ΔΔCt method and using normoxia as control condition. The *y*-axis is interrupted at value 1.0 indicating down-regulation of gene expression below and up-regulation above this value. ^∗^*p* < 0.05, ^∗∗^*p* < 0.01, ^∗∗∗^*p* < 0.001, and ^∗∗∗∗^*p* < 0.0001.

We analyzed relative gene transcript levels compared to the normoxic condition after 4, 24, and 72 h for cell adhesion molecules involved in the inflammatory response, members of the fibrinolytic system, stress response genes, and other markers for lung endothelial function (Figure 3). While ICAM1 expression had a downward trend at 4 h under intermittent hypoxia, it was unchanged under all conditions at 24 h and increased under all hypoxic conditions after 72 h with the highest level under intermittent hypoxia (fold change +6.7 ± 2.1, *p* < 0.0001). Similarly, VCAM1 had a slight downward trend at 4 h under intermittent hypoxia, was significantly down regulated after 24 h of anoxia (fold change 0.4 ± 0.1; *p* < 0.0001) and unchanged under intermittent hypoxia, was still downregulated at 72 h of anoxia (fold change 0.2 ± 0.2; *p* < 0.0001), but was to be upregulated under intermittent hypoxia after 72 h (fold change +1.5 ± 0.4; *p* < 0.001). p-Selectin expression slightly increased at 4 h under moderate hypoxia (+1.3 ± 0.2; *p* < 0.01), was especially low under severe hypoxia 0.3 ± 0.04-fold expression after 24 h (*p* < 0.001) and 0.2 ± 0.8-fold expression (*p* < 0.0001) after 72-h compared to normoxia, but its level was not or only slightly changed under intermittent hypoxia at all time points. NOS3 expression was statistically unchanged after 4 and 24 h under moderate and intermittent hypoxia. A strong effect was observed under severe hypoxia, with upregulation at 4 h (2.2 ± 0.8; *p* = 0.032) and downregulation at 72 h (0.27 ± 0.21; *p* < 0.01). Intermittent hypoxia induced a down regulation or trend at 4 h intermittent hypoxia for uPA, tPA, and PAI (fold change tPA 0.3 ± 0.4; *p* < 0.01) and a slight upregulation of uPA (+1.5 ± 0.5-fold change compared to normoxia; *p* = 0.020), tPA (+1.7 ± 0.6-fold change compared to normoxia; *p* < 0.01), and PAI (1.7 ± 0.6-fold change compared to normoxia; *p* < 0.01) after 72 h intermittent hypoxia. Endothelial activation results in the release of the Weibel-Palade bodies, which are storage granules of endothelial cells containing as major components p-selectin, vWF, and angiopoietin-2 (ANGPT2). vWF mRNA was downregulated after 4 h intermittent hypoxia (fold change 0.5 ± 0.2; *p* < 0.01) and slightly upregulated after 24 h (fold change 1.3 ± 0.3; *p* = 0.028) and after 72 h of intermittent hypoxia (+1.5 ± 0.4 fold change compared to normoxia; *p* < 0.001). ANGPT2 was upregulated under severe hypoxia (+2.1 ± 2.0 at 4 h; *p* = 0.035; +3.1 ± 0.5 fold change at 24 h; *p* < 0.0001 and +3.1 ± 0.8 fold change after 72 h compared to normoxia; *p* < 0.0001), but with a trend downregulated after 4 h (fold change 0.4 ± 0.12; n.s.), 24 h (fold change 0.78 ± 0.1; n.s.), and with a trend upregulated after 72 h of intermittent hypoxia (fold change +1.4 ± 0.5; n.s.). The inflammatory transcription factor NFκB subunit p65 showed no change at 4 h of all conditions, showed a trend toward upregulation under all hypoxic conditions at 24 and 72 h with high variation, but the strongest and statistically significant increase was observed with 72 h of intermittent hypoxia (+33.6 ± 10.31 fold change compared to normoxia; *p* < 0.0001). The cellular stress protein heme oxygenase-1 (HMOX1) was strongly upregulated under severe hypoxia (+6.3 ± 6.7 fold change after 4 h; *p* < 0.0001; +8.9 ± 1.9 fold change after 24 h; *p* < 0.0001 and +4.5 ± 1.5 fold change after 72 h compared to normoxia; *p* < 0.01). Intermittent hypoxia induced a trend to down-regulation of HMOX1 after 4 h (0.5 ± 0.1 fold change; n.s.), 24 h (0.7 ± 0.1 fold change compared to normoxia; n.s.), and no change after 72 h. Receptor for advanced glycation endproducts (RAGE) had a downward trend at 4 h intermittent hypoxia (fold change 0.3 ± 0.1 fold change; n.s.), was upregulated after 24 h under anoxia (+2.2 ± 0.9 fold change under intermittent hypoxia compared to normoxia; *p* < 0.001), and had also a slight upward trend after 24 and 72 h under intermittent hypoxia. Finally, the renin-angiotensin-system (RAS) representative angiotensin-converting enzyme 1 (ACE1) did not show any major changes apart from anoxia, where it was upregulated (+4.4 ± 3.1 fold change compared to normoxia; *p* < 0.0001).

### Chronic Intermittent Hypoxia Affects the Expression of Specific Proteins Involved in the Dissipation of Oxidative Stress

Using a qRT-PCR array, we measured the expression of 84 genes involved in the cellular management of oxidative stress (for a list of all tested genes see Supplementary Material). While most proteins were affected by continuous hypoxia (moderate hypoxia and anoxia), intermittent hypoxia selectively changed the expression of only five proteins with statistical significance compared to normoxia: glutathione peroxidase 1 was reduced 2.9-fold (*p* = 0.03), the superoxide dismutases 2 and 3 were reduced by 5.9 and 5.5-fold (both *p* < 0.001), respectively, thioredoxin 1 was reduced by 7.3-fold (*p* = 0.012), and the related thioredoxin reductase 3 was even reduced by 9.1-fold (*p* = 0.01) (Table 2). It is interesting to note, that the downregulation of SOD2, Txn1, and Txnrd3 was much stronger under intermittent hypoxia compared to constant moderate hypoxia or anoxia.

**Table 2:** Statistically significant changes of relative gene expression of proteins/enzymes involved in dissipation of oxidative stress under 72 h of intermittent hypoxia compared to moderate or severe hypoxia.

Gene	0% O_2_	10% O_2_	0–21% O_2_
Gpx1	n.s.	-2.4 (*p* < 0.01)	-2.9 (*p* = 0.003)
Gstp1	n.s.	n.s.	-24.2 (*p* = 0.037)
Serpb1b	n.s.	-3.1 (*p* < 0.01)	-5.4 (*p* = 0.0008)
SOD2	-2.9 (*p* < 0.01)	-2.7 (*p* < 0.01)	-5.9 (*p* = 0.0005)
SOD3	-6.0 (*p* < 0.01)	n.s.	-5.5 (*p* = 0.002)
Txn1	-1.3 (*p* < 0.05)	-3.8 (*p* < 0.05)	-7.3 (*p* = 0.012)
Trxrd3	+21.4 (*p* < 0.01)	n.s.	-9.1 (*p* = 0.011)


*Relative gene expression was quantified by qRT-PCR using the ΔΔCt method and using normoxia as control condition. *P*-values indicate statistical significance. n.s., not statistically significant.*

### Regulation of MicroRNA Expression Under Chronic Intermittent Hypoxia

Protein expression is not only regulated by transcription, but also by post-transcriptional mechanisms including small non-coding miRNAs. Dysregulated levels of microRNAs are frequently observed in pathological conditions and are often an indicator for the onset or progression of disease. Relative expression of 84 micro RNAs was quantified by qRT-PCR after 72 h of exposure to normoxia, constant moderate hypoxia and intermittent hypoxia. The abundance of three micro RNAs was selectively reduced under intermittent hypoxia: mmu-miR-21a-5p was reduced by 189.3-fold (*p* = 0.027), mmu-miR-322-5p was reduced by 36.5-fold (*p* = 0.048) and mmu-miR-218-5p was reduced by 19.8-fold (*p* = 0.004) compared to normoxia, while no significant change was observed under moderate hypoxia for these miRNAs (Table 3).

**Table 3:** Statistically significant changes of relative abundance of miRNAs under 72 h of intermittent hypoxia compared to moderate hypoxia.

miRNA	10% O_2_	0–21% O_2_
mmu-miR-21a-5p	n.s.	-189.3 (*p* = 0.027)
mmu-miR-322-5p	n.s.	-36.5 (*p* = 0.048)
mmu-miR-218-5p	n.s.	-19.8 (*p* < 0.004)


*Relative amounts of miRNAs were quantified by qRT-PCR using the ΔΔCt method and using normoxia as control condition. *P*-values indicate statistical significance.*

### Measured RNA Levels Are Reflected in Protein Levels

**FIGURE 4:**
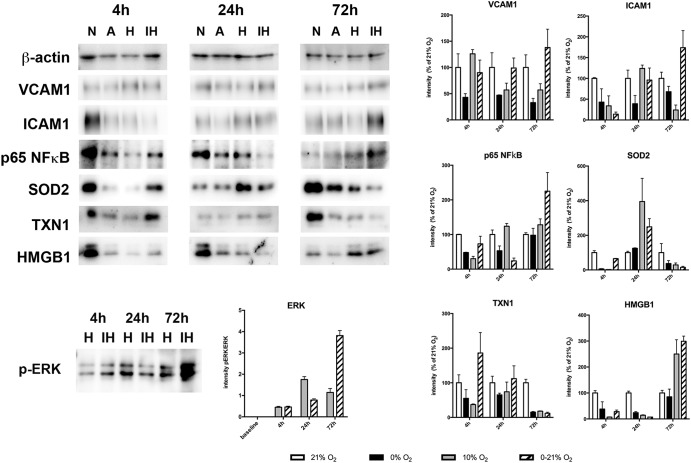
Western blot analysis of protein expression of VCAM1, ICAM1, p65NFκB, SOD2, TXN1, HMGB1, and phospho-ERK1/2 after 4, 24, and 72 h exposure to different gas conditions. N, normoxia (21% O_2_); A, anoxia (0% O_2_); H, moderate hypoxia (10% O_2_); IH, intermittent hypoxia (0–21% O_2_). Images show representative blots, graphs depict densitometric quantifications from three samples.

We assessed expression of selected proteins by Western blotting and detection with specific antibodies (Figure 4). Intermittent hypoxia resulted in constant levels of VCAM1 until 24 h (90 ± 24% and 99 ± 19% compared to normoxia levels at 4 and 24 h, respectively) and increased levels to 138 ± 35% at 72 h. ICAM1 exhibited low levels at 4 h of intermittent hypoxia (14 ± 5% compared to normoxia) and increased gradually until 72 h reaching 174 ± 41% of normoxic levels. NFκB p65 was lowest at 24 h (24 ± 8% compared to normoxia) and increased to 225 ± 54% at 72 h of intermittent hypoxia. SOD2 peaked at 24 h with 249 ± 47% compared to normoxia and plummeted to 15 ± 6% until 72 h. TXN1 reached 186 ± 59 early at 4 h and degraded thereafter to reach only 12 ± 3% of normoxia levels at 72 h of intermittent hypoxia. Finally, HMGB1 showed low expression levels until 24 h (28 ± 6% at 4 h and 7 ± 0.1% at 24 h) and increased after that to reach 299 ± 20% at 72 h of intermittent hypoxia.

Activation of the MAPkinase ERK1/2 by sustained moderate hypoxia (10% O_2_) and intermittent hypoxia (0–21% O_2_) was reflected by an increase of the phosphorylation state of (Thr202/Tyr204). Phosphorylated ERK (normalized to total ERK protein) increased threefold to a plateau level at 24 h under sustained hypoxia, while intermittent hypoxia continuously increased phosphorylated ERK until 72 h to reach approximately ninefold of baseline levels.

### Functional Aspects: Chronic Intermittent Hypoxia Affects Endothelial Monolayer Permeability

**FIGURE 5:**
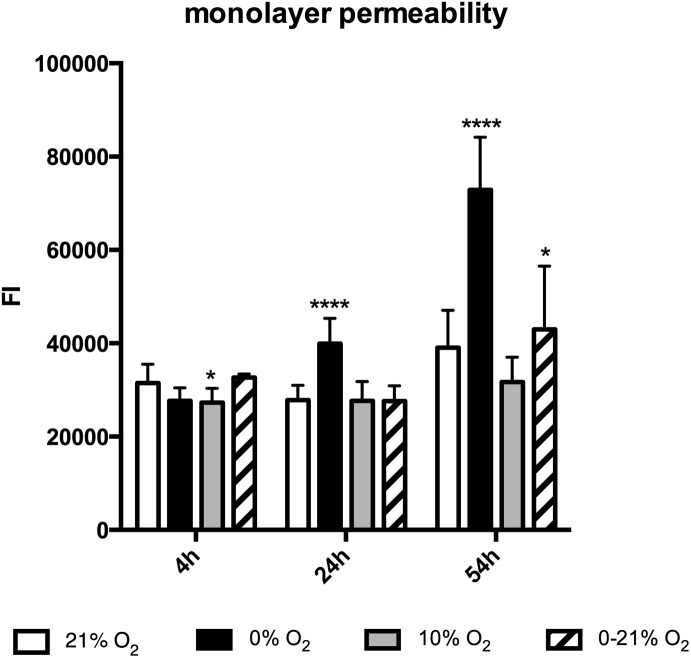
Monolayer permeability after 4, 24, and 72 h exposure to different gas conditions. The *y*-axis depicts fluorescence intensity units (FI). ^∗^*p* < 0.05; ^∗∗∗∗^*p* < 0.0001.

Confluent cultures of mouse lung endothelial cells were exposed to normoxia, moderate hypoxia, anoxia and intermittent hypoxia and after 4, 24, and 54 h the accessibility of the coated gelatine for a fluorophore-labeled ligand given to the medium was assessed by reading the emitted fluorescent light from the bottom of the plate (Figure 5). After 4 h exposure the permeability of the layer was lowest under moderate hypoxia (27.267 ± 3.088 FI compared to normoxia: 31.464 ± 4.021 FI; *p* < 0.05). At 24 h the permeability had markedly increased under severe hypoxia (39.922 ± 5.410 FI versus 27.820 ± 3.182 FI under normoxia; *p* < 0.0001). At 54 h the permeability had further increased under severe hypoxia and also reached a significant level under intermittent hypoxia (42.963 ± 13.549 FI under intermittent hypoxia versus 31.677 ± 5.320 FI under moderate hypoxia and 39.038 ± 8.031 FI under normoxia; *p* < 0.05).

## Discussion

Intermittent hypoxia is known to contribute to the detrimental consequences of complex conditions like obstructive sleep apnea syndrome or cyclic recruitment and derecruitment of atelectasis in acute lung injury. Our study aimed to investigate short- and long-term effects of intermittent hypoxia on isolated lung endothelial cells in order to identify molecular mechanisms that contribute to the pathology and could be targets for future therapeutical interventions. Our results show, that short-term (4–24 h) exposure to moderate frequency intermittent hypoxia is seemingly well tolerated without significant loss in vitality or major occurrence of indicators for injury. On the contrary, chronic exposure to IH elaborates multiple ways of inflammatory and injurious processes, every single one of those being capable of inducing cellular damage.

Cell growth and secreted factors represent the overall outcome parameter for the sum of molecular events: While 24 h of intermittent hypoxia is depicted by normal cell growth and absence of significant increase in inflammatory cytokines, 72 h of intermittent hypoxia reveals the detrimental effects: cell numbers are reduced by around 45% and a significant release of inflammatory cytokines like IL6 and CXCL1 is detected. Further markers of inflammatory processes are the increase of cell adhesion molecules ICAM1 and VCAM1 after 72 h. Again, these markers are not elevated after 24 h, but on the contrary are even down-regulated at earlier timepoints. Components of the fibrinolytic system uPA, tPA, and PAI-1 are changed after 72 h of intermittent hypoxia, resulting in an upregulation of uPA and tPA and PAI1. Plasminogen activators might have beneficial effects on fibrin deposition and associated inflammation, but on the other side tPA knockout mice have revealed especially positive effects with regard to decreased vascular permeability and improved lung function including pulmonary artery pressure, airway resistance, and pulmonary compliance (Zhao et al., 2011).

An overactive renin-angiotensin-system (RAS) in the lung can boost the inflammatory factors ICAM, VCAM, TNFα, and IL6 and can have profibrotic effects (Pacurari et al., 2014). In our experiments, we observed upregulation of ACE1 under severe hypoxia after 72 h. Intermittent hypoxia revealed almost no change at all timepoints. An indication of higher risk with regard to coagulation and thrombogenesis might be the increase of vWF levels under intermittent hypoxia after 24 and 72 h. At 4 h this factor is downregulated under intermittent hypoxia and upregulated at later timepoints. Together with ANGPT2, vWF is frequently regarded as a marker for endothelial damage and higher soluble plasma levels are correlated with increased mortality in acute lung injury (Calfee et al., 2012). ANGPT2 levels were down after 4 h and unchanged after 72 h under intermittent hypoxia.

An indicator for strong proinflammatory processes under long-term intermittent hypoxia is the exuberant increase of NFκB subunit p65 after 72 h, and this is consistent with the increase of proinflammatory cytokines at this time point, although the major regulation of the activity of this transcription factor is done post-translationally. Induction of p65 mRNA expression can be mediated by the pattern recognition receptor (PRR) RAGE that becomes activated due to tissue damage and endothelial activation (Kierdorf and Fritz, 2013). RAGE has its highest expression levels in the lung, where in contrast to other areas it is regarded as a protective factor, as down-regulation is observed in several pathological manifestations including lung tumors. RAGE is also lost in the lungs of patients with idiopathic pulmonary fibrosis and this down-regulation can be elicited by application of inflammatory cytokines to lung fibroblasts and epithelial cells *in vitro* (Queisser et al., 2008). After 4 h of intermittent hypoxia RAGE mRNA is down-regulated probably as an anti-inflammatory cellular measure. Down-regulation of RAGE might be mediated by active repression, post-transcriptional, or epigenetic mechanisms. It has been shown, that the 3′ UTR of the RAGE gene contains several signals that affect mRNA stability and turn over that might quickly adapt cellular mRNA levels (Caballero et al., 2004). Under chronic intermittent hypoxia RAGE mRNA is upregulated again. This might be due to gene transcription via the NFκB and SP1 transcription factor binding sites in the RAGE promoter region, that are also responsive to HMGB1 (Fiuza et al., 2003). RAGE activation further increases its own expression representing a positive feedback loop due to ligand binding. Intracellular signaling pathways downstream of RAGE activation include small GTPases, MAPkinases, NFκB, and CREB. MAPkinase ERK1/2 activation as observed in our setting might be initially accomplished by ROS generation due to intermittent hypoxia and might be further boosted by a later increase of RAGE activation. Activated ERK1/2 is usually considered a “pro-survival” kinase. However, other detrimental cellular events might override this signal.

Finally, the cellular stress protein HMOX1 is already especially upregulated under severe hypoxia at early timepoints. HMOX1 is frequently regarded as a target for treatment of inflammatory conditions, as this enzyme degrades heme to biologically active end products that affect apoptosis and inflammation (Ryter and Choi, 2016). We observed a slight downregulation of HMOX1 after 4 h intermittent hypoxia returning to baseline at later timepoints.

It is interesting to note, that while constant hypoxia affects many cellular redox systems, chronic intermittent hypoxia selectively impacts GPX1, Gstp1, SOD2, SOD3, Txn1, and Txnrd3, each with a strong downregulation after 72 h. GPX1 deficiency has been shown to facilitate inflammation and activation of the vascular endothelium (Sharma et al., 2016). Similarly, inactivation of Txn1 in a mouse transgene model resulted in increased levels of proinflammatory cytokines (Das, 2015). Gstp1 is an enzyme, that catalyses *S*-glutathionylation of proteins (frequently under conditions of oxidative stress), thus ultimately leading to functional changes. One important target is IKKβ, a kinase involved in the activation of the proinflammatory NFκB pathway. Sufficient catalytic activity of Gstp1 is vital for repressing NFκB. Downregulation of Gstp1 leads to reduced *S*-glutathionylated IKKβ and an increased nuclear translocation of NFκB with a concomitant increase of inflammatory cytokines (Jones et al., 2016). Finally, the SODs are principal protective enzymes that have a key importance in the detoxification of superoxide radicals. The extracellular SOD3 has crucial functions especially in the endothelium. SOD3 maintains NO bioavailability and protects against several lung disorders, including oxidative injury, emphysema, inflammation, and fibrosis (Yao et al., 2010; Ahmed et al., 2012). Overexpression of SOD3 has been shown to attenuate hypoxic pulmonary vascular remodeling and to preserve angiogenesis in the lung that is halted by oxidative stress (Perveen et al., 2012). Animal models of intermittent hypoxia have shown that epigenetic mechanisms like DNA methylation can regulate expression of isoforms of antioxidative genes SOD, Prdx, GPX, and Txnrd in a tissue and cell-specific way upon expression changes of DNA methylating enzymes (Nanduri et al., 2017). A more direct mechanism of downregulation of antioxidative enzymes is via downregulation of their transcription factor HIF2α via calcium-dependent calpains (Nanduri et al., 2009). Calcium signaling underlies also intermittent hypoxia-induced upregulation of the HIF2α ortholog HIF1α, that has been investigated into detail in this context. ROS generation and subsequent Ca^2+^ mobilization by intermittent hypoxia has been shown to be mediated by an upregulation of NADPH oxidase subunits p47^phox^ and p67^phox^, ROS-dependent activation of PLCγ and IP3/DAG generation. Subsequent HIF1α upregulation is accomplished by a decrease in proline hydroxylation and therefore decreased degradation via the proteasome and at the same time by a Ca^2+^ dependent activation of mammalian target of rapamycin (mTOR) and enhanced protein synthesis. Calcium-dependent kinases (CaMKs) additionally phosphorylate the co-activator p300 in order to enhance HIF1α transcriptional activity (Yuan et al., 2005, 2008). Tofolli et al. (2007) observed an additional specific phosphorylation of HIF1α by the kinase PKA in endothelial cell lines under low-frequency intermittent hypoxia, that seemed to enhance transcriptional activity.

ROS have a central role in the effects of intermittent hypoxia: They might be subtle regulators of enzyme function in the early periods, but the oscillating oxygen conditions in the longer term will let accumulate ROS from various sources like mitochondria, peroxisomes, lysosomes and endoplasmic reticulum to overall toxic levels. In addition, down-regulation of antioxidant enzymes through multiple mechanisms adds to the cellular oxidative stress.

Finally, we investigated the impact of intermittent hypoxia on the activity of subtle gene expression regulators, the miRNAs. miRNAs are 20–22 nucleotide RNA molecules involved in gene expression regulation. They are frequently regarded as biomarkers for certain disease states and are also potential therapeutic targets as their expression can be modified by various strategies like the use of antagomiRs, miRNA sponges and mimetics. The operation mode of miRNAs is very often not straight forward, as single miRNAs can regulate many different mRNAs and many potential targets can only be predicted by bioinformatic algorithms but are not validated experimentally yet. We analyzed changes of miRNA expression under chronic intermittent hypoxia revealing a strong reduction of mmu-miRNA-21a-5p, mmu-miRNA-322, and mmu-mir-148. miR-21a is known for its anti-apoptotic properties. Examples for validated targets of miR-21a are Fasl, Pdcd4, Pten, Smad7, Spry, Bcl2, Mmp9, and Sod2^1^. A downregulation of miRNA-21 could indicate that pro-apoptotic molecules like Fasl and Pten are less suppressed. There is also evidence, that downregulation of miR-21 has beneficial effects on lung compliance and barrier function (Vaporidi et al., 2012). Mmu-mir-322 down-regulation has been described in the context of endoplasmic reticulum (ER) stress, the unfolded protein response (UPR), and the related ER Ca^2+^ depletion (Groenendyk et al., 2014). Another study reports the involvement of miR-322 (or the human ortholog hsa-mir-424) in the protection from hypoxia-induced apoptosis (Yang et al., 2016). The expression of miR-322 by itself is regulated by HIF1 representing an important mechanism in pulmonary vascular remodeling in pulmonary hypertension (Zeng et al., 2015). Upregulation of mmu-mir-322/hsa-mir-424 has been observed in the pathological proliferation of vascular smooth muscle cells and endothelial cells under ischemic injury.

Finally, a forced downregulation of mmu-mir-218 has been shown to protect against intermittent hypoxia-induced apoptosis (Liu et al., 2017). Though, the authors describe upregulation of this miRNA as a response to IH in mice. An interesting feature of miR-218 is its target HMGB1. HMGB1 is frequently elevated in pathological conditions like sepsis or cancer and it is therefore regarded as damage-associated pattern (DAMP). HMGB1 is an agonistic ligand of RAGE that frequently activates proinflammatory genes (via activation of NFκB) and is elevated in chronic disease resulting from vascular damage. Gu et al. (2016) observed upregulation of HMGB1 upon down-regulation of miR-218 in gliomas, a feature that we also detected in our lung cells by immunoblotting. Upregulated HMGB1 will contribute to the disruption of endothelial barrier function as HMGB1 has been shown to down regulate adherence and tight junction proteins VE-cadherin and ZO-1 in lung endothelial cells (Luan et al., 2018). HMGB1 has also been shown in human microvascular endothelial cells to directly increase expression of ICAM1, VCAM1, TNFα, IL8, PAI1, and tPA (Fiuza et al., 2003).

Monolayer permeability as measured with our functional assay will be a surrogate of many factors including subtle intracellular changes up to major events like cell death. Makarenko et al. (2014) identified intermittent hypoxia ROS-activated MAPkinases ERK and JNK as crucial triggers to endothelial barrier compromise by mediating reorganization of the cytoskeleton and redistribution of junctional proteins. However, their model differs from our system in several aspects. They used human lung microvascular endothelial cells and their intermittent hypoxia cycles only comprised 30 s of hypoxia (1.5% O_2_). Barrier function was assessed by transendothelial electrical resistance (TEER) measurements as opposed to transmigration of a labeled avidin molecule in our assay. These factors might explain differences in the time onset of barrier dysfunction.

The effect of intermittent hypoxia on different organ systems has been observed in several animal experiments, sometimes with contradictory outcome. The available literature, however, draws its conclusions from diverse model systems especially with regard to intermittent hypoxia regimen. Generally, it can be assumed, that lower frequency and mild hypoxia as well as acute incidences more likely lead to adaptation of the organism and beneficial outcomes than high frequency oscillations with severe hypoxia and chronic exposure (Almendros et al., 2014). The beneficial effect of intermittent hypoxia is well known in the context of preconditioning of the heart and also training of athletes. A janus (beneficial/harmful) effect has been also observed in a mouse model of apnea of prematurity (AOP), where it was shown, that moderate intermittent hypoxia can stimulate neurogenesis and confer neuroprotection, while inducing neurodevelopmental disorders and apoptosis under more severe conditions (Bouslama et al., 2015). Similarly, low-frequency intermittent hypoxia as post-conditioning stimulus was shown to decrease infarct volume after brain ischemia together with improved learning and memory performances (Tsai et al., 2011). The lung has also been shown to be a target organ for preconditioning by intermittent hypoxia. Zhang et al. (2004, 2009) showed, that moderate regimen of intermittent hypoxia can improve lung function with regard to gas exchange, barrier function and survival under hypoxic conditions in mice, especially by protecting hypoxia-sensitive alveolar type I epithelial cells. A higher frequency protocol of intermittent hypoxia, however, was shown to deteriorate lung fibrosis in a bleomycin mouse model including higher inflammation and mortality (Gille et al., 2017). Obstructive sleep apnea syndrome occurs with high prevalence in patients with idiopathic pulmonary fibrosis (IPF).

### Limitations of Our *in vitro* Study

In our study, we expose lung endothelial cells to intermittent hypoxia of moderate frequency, but full amplitude (0–21% O_2_). A major limitation of our study is the use of mixed lung endothelial cell types, a draw-back, that cannot be easily circumvented, as not special cell markers are known, that can distinguish between different phenotypes of endothelial cells in the lung. Also, cell culture media components might influence the expression of some endothelial markers, as for example described for heparin, ECGF and PAI expression (Konkle and Ginsburg, 1988). In order to tackle this problem, we kept the composition of the medium constant during each experiment. Therefore, relative changes of gene expression under different gas conditions are with a very high probability observed due to the influence of oxygen. Another issue is the frequency of O_2_-oscillations for intermittent hypoxia that is limited in our system due to technical reasons. A recent study tested several frequencies and found variations in outcome (Campillo et al., 2017). Our bioreactor has several advantages as to a direct transmission of O_2_-oscillations to the cells in the absence of shear stress. Yet, oscillation frequencies are limited by the time the gas needs to penetrate the membrane. Six cycles per hour are the maximum we can reach in our system verified to reach the cells in full amplitude.

We also state, that our approach was not designed to detect many early events exemplified by protein phosphorylation of multiple putative targets. A phospho-proteomic approach would be a suitable method to elucidate these signaling events and to further delineate mechanisms involved in protective or detrimental effects of intermittent hypoxia in specific cell types.

## Conclusion

In our study, we show that intermittent hypoxia of moderate frequency lasting for a few hours does not have immediate negative effect on lung endothelial cells. Some injury markers show even a reversed expression at 4 h, in some cases until 24 h. Our results are in accordance with the generally discussed hypothesis, that short-term lower frequency intermittent hypoxia can have a protective effect on cells in the sense of a preconditioning stimulus and this seems also to be true for lung endothelial cells. However, in the long-term, several factors unite to form a state of inflammation, to compromise barrier function, and to facilitate apoptosis. Knowing the specific pathways that are activated in individual cell types will definitely improve our understanding of what is actually going on in the whole organ and will help to develop tailor-made therapies that address these specific processes. With this aim, we argue that further detailed analyses of signaling pathways in response to intermittent hypoxia by including (phospho)-proteomic, epigenetic, and systems biology methodology are still desirable goals.

## Author Contributions

PW, KM, VT, and KK designed the study. PW, LS-G, and VT performed the study. MW and IL provided the expertise in cell culture and lung endothelium. PW, VT, TB, and KK analyzed the data. VT and KK wrote the manuscript.

## Conflict of Interest Statement

The authors declare that the research was conducted in the absence of any commercial or financial relationships that could be construed as a potential conflict of interest.

## Footnotes

1 http://mirtarbase.mbc.nctu.edu.tw

## Supplementary Material

FIGURE S1 List of all genes investigated in the mouse oxidative stress PCR array (Qiagen, Netherlands) by qRT-PCR.

FIGURE S2 List of all miRNAs investigated in the miRNA PCR Array Mouse miFinder (Qiagen, Netherlands).

## Abbreviations

ACE: angiotensin converting enzyme
ANGPT: angiopoietin
Bcl: b-cell lymphoma
CXCL: chemokine (C-X-C motif) ligand
ECGF: endothelial cell growth factor
ERK: extracellular signal-regulated kinase
Fasl: Fas ligand
GPX: glutathione peroxidase
Gstp: glutathione-S-transferase pi
HIF: hypoxia-inducible factor
HMGB: high mobility group protein
HMOX: heme oxygenase
ICAM: intercellular adhesion molecule
IKK: inhibitor of nuclear factor kappa B kinase
IL: interleukin
IP3/DAG: inositol-3-phosphate/diacyl glycerol
JNK: c-Jun N-terminal kinase
MAPkinase: mitogen-activated protein kinase
MIP: macrophage inflammatory protein
Mmp: metallo-proteinase
NFκB: nuclear factor kappa-light-chain-enhancer of activated B-cells
NOS: nitric oxide synthase
PAI: plasminogen activator inhibitor
Pdcd: programmed cell death protein
PLC: phospholipase C
Pten: phosphatase and tensin homolog
RAGE: receptor for advanced glycation endproducts
SOD: superoxide dismutase
Spry: sprouty homolog
TNFα: tumor necrosis factor alpha
tPA: tissue plasminogen activator
Txn: thioredoxin
Txnrd: thioredoxin reductase
uPA: urokinase-type plasminogen activator
VCAM: vascular cell adhesion protein
VE-cadherin: vascular endothelial cadherin
VEGF: vascular endothelial growth factor
TNFα: tumor necrosis factor alpha
vWf: Von Willebrand factor
ZO: zonula occludens
