# Supplementary figures and images for: Intermittent Hypoxia Activates Duration-Dependent Protective and Injurious Mechanisms in Mouse Lung Endothelial Cells
Source: Front Physiol. 2018 Dec 6;9:1754. doi: 10.3389/fphys.2018.01754 (PMC6291480; doi:10.3389/fphys.2018.01754)

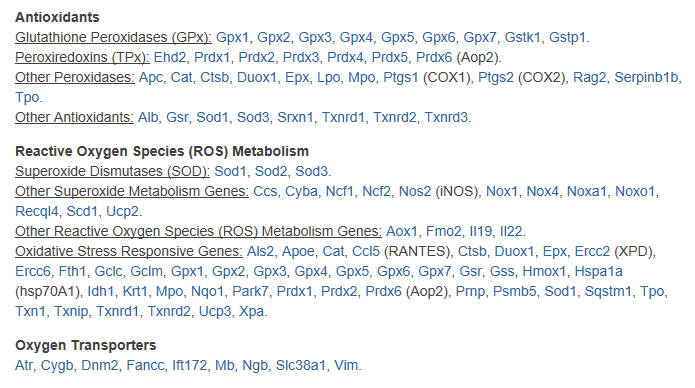

Supplement: FIGURE S1 — List of all genes investigated in the mouse oxidative stress PCR array (Qiagen, Netherlands) by qRT-PCR. [file Image_1.TIF]

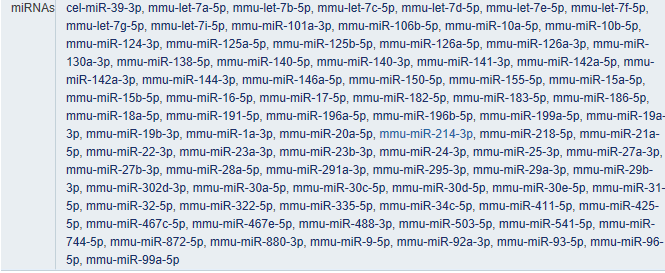

Supplement: FIGURE S2 — List of all miRNAs investigated in the miRNA PCR Array Mouse miFinder (Qiagen, Netherlands). [file Image_2.TIF]
